# Latent HIV-1 TAR Regulates 7SK-responsive P-TEFb Target Genes and Targets Cellular Immune Responses in the Absence of Tat

**DOI:** 10.1016/j.gpb.2017.05.003

**Published:** 2017-10-14

**Authors:** Sebastian Eilebrecht, Bernd-Joachim Benecke, Arndt G. Benecke

**Affiliations:** 1 CNRS UMR8246, Université Pierre et Marie Curie, Paris 75005, France; 2 ACSIOMA GmbH, Technologiezentrum Ruhr, Bochum 44799, Germany; 3 Center for Innate Immunity and Immune Disease, University of Washington School of Medicine, Seattle, WA 98195, USA

**Keywords:** HIV-1, Latency, TAR, 7SK RNA, Cellular immunity

## Abstract

The transactivating response element (**TAR**) structure of the nascent **HIV-1** transcript is critically involved in the recruitment of inactive positive transcription elongation factor b (P-TEFb) to the promoter proximal paused RNA polymerase II. The viral transactivator Tat is responsible for subsequent P-TEFb activation in order to start efficient viral transcription elongation. In the absence of the viral transactivator of transcription (Tat), *e.g.*, during **latency** or in early stages of HIV transcription, TAR mediates an interaction of P-TEFb with its inhibitor hexamethylene bis-acetamide-inducible protein 1 (HEXIM1), keeping P-TEFb in its inactive form. In this study, we address the function of HIV-1 TAR in the absence of Tat by analyzing consequences of HIV-1 TAR overexpression on host cellular gene expression. An RNA chimera consisting of Epstein-Barr virus-expressed RNA 2 (EBER2) and HIV-1 TAR was developed to assure robust overexpression of TAR in HEK293 cells. The overexpression results in differential expression of more than 800 human genes. A significant proportion of these genes is involved in the suppression of cellular immune responses, including a significant set of 7SK-responsive P-TEFb target genes. Our findings identify a novel role for HIV-1 TAR in the absence of Tat, involving the interference with host cellular immune responses by targeting **7SK RNA**-mediated gene expression and P-TEFb inactivation.

## Introduction

HIV-1 latency is established by an interplay of different mechanisms, which render the HIV provirus transcriptionally silent. A major step for the activation of HIV-1 gene expression is the switch from transcriptional stalling to productive transcription elongation. The HIV-1 core promoter contains binding sites for a number of different transcription factors, such as nuclear factor of activated T cells (NFAT), nuclear factor kappaB (NF-κB), and specificity protein 1 (SP1) [1], [2], [3], which are involved in the regulation of viral transcription. Upon initial transcription initiation, RNA polymerase II (RNAPII) produces a nascent viral transcript, before it is stalled proximal to the transcription start site (TSS) due to its non-processive phosphorylation status as well as the transcription inhibitory activities of 5,6-dichloro-1-β-d-ribofuranosylbenzimidazole (DRB) sensitivity–inducing factor (DSIF) and negative elongation factor (NELF) [4], [5], [6]. The nascent transcript forms a stable hairpin structure, the transactivating response element (TAR), which serves as a binding platform for a number of cellular and viral proteins [7], [8], [9], [10].

An essential host cellular factor for the switch toward productive transcription elongation is the positive transcription elongation factor b (P-TEFb), whose active form consists of the cyclin-dependent kinase 9 (CDK9) and one of the cyclins, T1 or T2 [11], [12]. Active P-TEFb phosphorylates the carboxy-terminal domain (CTD) of RNAPII, as well as DSIF and NELF, turning DSIF into an elongation-promoting factor and releasing NELF from the stalled RNAPII [13], [14]. A large proportion of cellular P-TEFb is sequestered in an inactive complex with the hexamethylene bis-acetamide-inducible proteins 1 or 2 (HEXIM1/2), the COUP-TF interacting protein 2 (CTIP2) and the small nuclear RNA (snRNA) 7SK [15], [16], [17]. We have recently shown that the inactive P-TEFb complex is recruited to the HIV-1 core promoter by the high mobility group protein A1 (HMGA1) that interacts with 7SK [18], [19], [20], [21], [22]. As soon as sufficient amounts of the viral transactivator of transcription (Tat) are expressed, the protein is able to release 7SK and HEXIM1/2 from the inactive complex, resulting in P-TEFb activation [23], [24]. Subsequently, the resulting active Tat/P-TEFb complex is recruited to the promoter proximal paused RNAPII by specific interactions with TAR [13], [25], thus initiating the efficient viral transcription elongation reaction. Sedore and co-workers have previously shown that P-TEFb and HEXIM1 bind to TAR in the absence of Tat [26]. In this case, P-TEFb is inactive due to the inhibitory function of the HEXMI1 protein present.

Apart from that, TAR has a number of additional cellular interacting partners, such as the TAR RNA binding protein (TRBP), HMGA1, or the La auto-antigen [7], [8], [9]. These interactions point at additional functions of TAR besides HIV-1 transactivation. In this study, we try to examine such functions of HIV-1 TAR, utilizing heterologous fusions with the Epstein-Barr virus-expressed RNA 2 (EBER2) [27]. Similar strategies have previously been proven effective for investigating the HMGA1 regulation by 7SK L2 substructures [18]. Here we show that heterologous TAR expression in the absence of Tat results in significant changes in the expression of host genes involved in immune regulation. These modulating activities can be ascribed to interference with 7SK and P-TEFb mediated gene expression, which would contribute to a better understanding of HIV-1 latency and early reactivation.

## Results and discussion

### HIV-1 TAR can be efficiently expressed as EBER2 RNA chimera

*EBER2* is a non-coding nuclear RNA transcribed by RNAPIII. About 10^6^–10^7^ copies of *EBER2* can be detected in one EBV-infected host cell [27], [28], making the *EBER2* promoter highly attractive for overexpression of small RNA structures [18], [29], [30]. Previous studies have quantified the levels of multiply spliced viral mRNAs during HIV-1 proviral latency in peripheral blood mononuclear cells (PBMCs) from HIV-1-seropositive patients. These mRNAs could amount to about 5 × 10^3^ copies per PBMC [31] and about 1 × 10^4^ copies per CD4^+^ T cell, which represent the major reservoir of latent HIV-1 in the blood and account for about 40% of the PBMCs [32]. During reactivation, Tat induces efficient HIV-1 transcription by a factor of approximately 10–60 [19], [33], resulting in up to 6 × 10^5^ RNA molecules per infected cell. Thus, the use of the EBER2 promoter-driven HIV-1 TAR chimera would confer a robust 100–1000-fold overexpression as compared to the latency state and a 2–20-fold overexpression as compared to the state of reactivation. Such overexpression should be sufficient to efficiently titrate potential binding factors and obtain a maximal downstream effect on gene expression.

**Figure 1:**
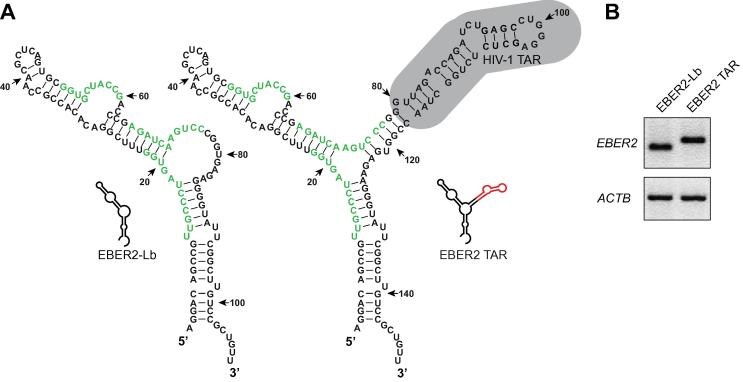
**Schematic representation and expression of EBER2 TAR fusion RNAs** **A.** Schematic representation of the EBER2-TAR fusion transcript for the expression of HIV-1 TAR RNA structure (EBER2 TAR). The second hairpin (loop b, Lb) of wild type EBER2 RNA of Epstein-Barr virus was replaced with the terminal hairpin structure of HIV-1 TAR (highlighted in gray and in red in the schematic structure). EBER2-Lb, the EBER2 backbone lacking the Lb structure completely, was used as a control. Gene internal promoter elements of the EBER2 backbone are highlighted in green. Nucleotide numbers are indicated. **B.** Expression of EBER2-Lb and EBER2 TAR in HEK293 cells. HEK293 cells were transfected with the constructs EBER2-Lb or EBER2 TAR. Expression levels of both constructs were monitored 48 h post transfection by RT-PCR. *ACTB* was used as a reference gene.

We have previously utilized the EBER2 promoter to overexpress the HMGA1-binding 7SK L2 substructure in the HEK293 cell line, resulting in the regulation of the vast majority of cellular HMGA1 target genes [18]. Besides gene-external promoter elements such as an Sp1 site, an activating transcription factor (ATF)-binding site, and the EBER TATA box (ETAB), the *EBER2* promoter also consists of gene internal box A and box B elements. Given these elements are essential for promoter activity, an EBER2 backbone has to be kept for RNA fusion approaches [34] (Figure 1A). Moreover, expression of this EBER2 backbone has to be used as an appropriate negative control in transcriptome analyses in order to identify specific target genes for the RNA structure of interest.

The 172-nt-long EBER2 RNA folds into a Y-like structure with two stem-loops, named loop a (La) and loop b (Lb), attached to a basal hairpin. The gene-internal promoter elements are located in La, which ends at nt 74, suggesting the exchange of Lb by the RNA of interest. Therefore, based on our positive experiences with the 7SK L2 chimeras [29], [34], we generated an EBER2-HIV-1 TAR chimeric RNA by replacing EBER2 Lb with nt 10 to 49 of HIV-1 TAR RNA. RNA secondary structure prediction suggested that both the EBER2 and the HIV-1 TAR part of the resulting chimeric construct fold similar to each RNA in its wild type context (Figure1A). Importantly, the EBER2-Lb construct lacking the EBER2 Lb structure completely, which was used as the EBER2 backbone negative control in these studies, folds the same way as in the context of wild type EBER2 (Figure 1A).

We next verified the transcriptional activity of the generated EBER2 TAR construct by semi-quantitative RT-PCR (Figure1B). We have shown previously that the EBER2-Lb control construct is transcribed at comparable levels as the wild type EBER2 RNA [29]. Also EBER2 RNA chimeras containing the HMGA1-binding 7SK L2 RNA substructure have shown a robust expression in HEK293 cells [18], [19]. Using *ACTB* (encoding beta actin) as a control, semi-quantitative measurements of the EBER2 TAR construct revealed no significant differences in transcription rates when compared to the EBER2-Lb construct (Figure1B). Notably, expression of HIV-1 TAR as an EBER2 chimeric RNA enables TAR expression without the context of the entire HIV-1 genome, ensuring that no HIV-1 Tat can be produced due to the lack of Tat coding sequence.

### HIV-1 TAR induced host cellular gene expression changes

**Figure 2:**
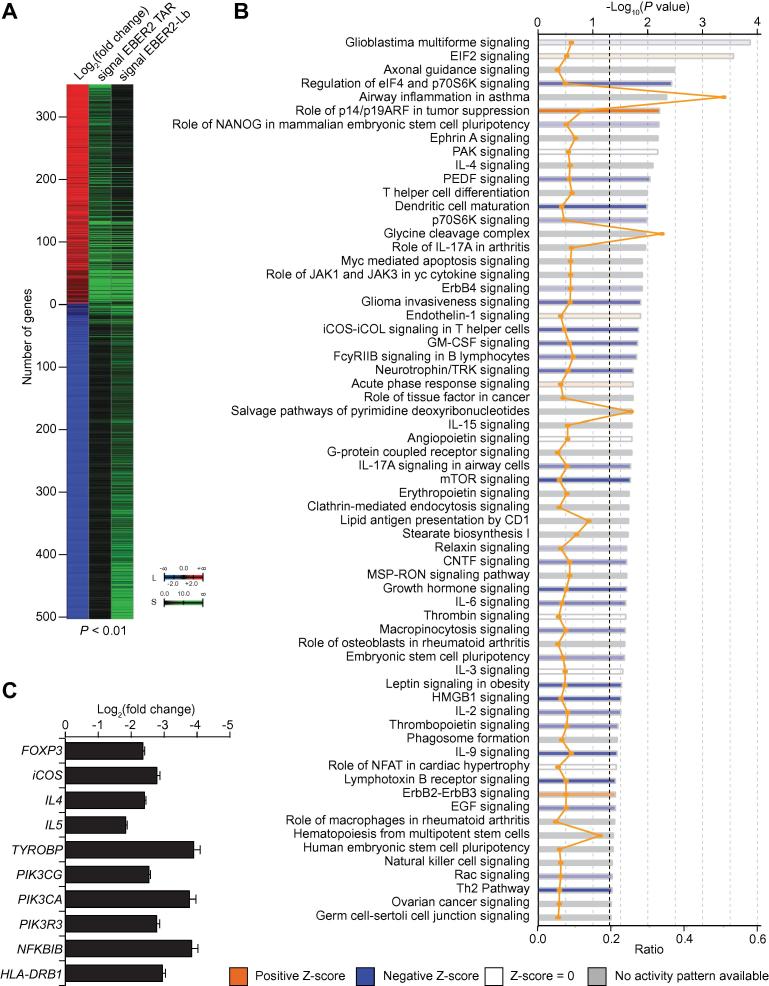
**Identification of HIV-1 TAR target genes in HEK293 cells** **A.** Heatmap of significant DEGs upon expression of the EBER2 TAR construct as compared to the expression of EBER2-Lb (*P* < 0.01). The log_2_ fold change (L) as well as the merged signals (S) of three biological replicates for each condition are indicated as a color scale. **B.** Pathway enrichment analyses for genes indicated in panel A. Significantly-enriched pathways are indicated together with the corresponding *P* values (bars) and ratios of the number of significantly-regulated genes per total number of genes involved in each pathway (orange line). The *P* value threshold (0.05) is indicated as dotted line. z-scores were calculated using Ingenuity Pathway Analysis. Negative z-scores (blue) indicate a repression and positive z-scores (orange) indicate an activation of the corresponding pathway. **C.** Log_2_ Fold changes in expression of immune-related genes upon EBER2 TAR expression. DEG, differentially-expressed gene.

In order to analyze the effect of HIV-1 TAR on the host cellular transcriptome in the absence of Tat, we identified HIV-1 TAR target genes in HEK293 cells overexpressing HIV-1 TAR. As mentioned above, major host cellular players involved in HIV gene expression and latency, such as CDK9, 7SK, and HMGA1, have been extensively characterized previously in HEK293 cell line with respect to their regulatory functions [18], [29], [30], [35]. The expression of the EBER2 HIV-1 TAR chimera in HEK293 cells results in the significantly differential regulation of 859 cellular genes (*P* < 0.01), of which expression of 352 (41%) genes is up-regulated and expression of 507 (59%) genes is down-regulated, when compared to HEK293 cells expressing the EBER2-Lb control construct (Figure 2A and Table S1).

**Figure 3:**
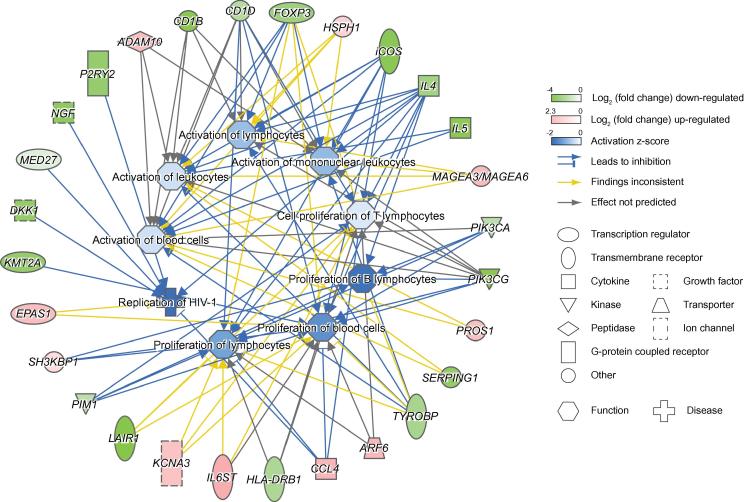
**HIV-1 TAR targets the cellular immune response in HEK293 cells** Genes regulated by HIV-1 TAR, which are involved in the repression of activation and proliferation of blood cells and in the repression of HIV-1 replication. Genes repressed and upregulated by TAR are highlighted in green and red, respectively, with the corresponding log_2_ fold change values given in color gradients. Genes are grouped according to their function by symbols. Blue arrows indicate repressive roles, inconsistent regulatory roles are indicated by yellow arrows, whereas gray arrows represent effects that are not predicted. Repressed pathways are indicated in blue, with the corresponding activation z-scores given in a color gradient.

Pathway analysis revealed several cellular pathways significantly enriched for these HIV-1 TAR target genes. These include T helper cell differentiation, IL-4 signaling, lipid antigen representation by CD1, inducible T-cell costimulatory (iCOS)-iCOS ligand (iCOSL) signaling in T helper cells, JAK1 and JAK3 in cytokine signaling, low affinity immunoglobulin gamma Fc region receptor II-b (FcgRIIb) signaling in B lymphocytes, IL-15 signaling, and granulocyte–macrophage colony-stimulating factor (GM-CSF) signaling, all of which are involved in cellular immune responses (Figure2B). A subset of these genes collaboratively suppress the activation of leukocytes and lymphocytes (Figure2C). The shRNA-mediated knockdown of *FOXP3*, which is significantly down-regulated by HIV-1 TAR expression (log_2_ fold change =  −2.94), has been shown to suppress the activity of naive human T-regulatory cells [36]. iCOS, encoded by another HIV-1 TAR suppressed gene, mediates the activation of T lymphocytes [37]. The interleukins IL-4 and IL-5, both of which are down-regulated by HIV-1 TAR, increase the activation of human lymphocytes and eosinophils, respectively [38], [39]. The TYRO protein tyrosine kinase-binding protein encoded by *TYROBP*, a HIV-1 TAR-suppressed gene, mediates the activation of natural killer cells together with natural cytotoxicity triggering receptor 2 (NCR2) [40]. All mentioned genes are down-regulated upon EBER2 HIV-1 TAR overexpression and are part of a regulated gene network, which results in a global repression of immune cell activation and proliferation, as well as an inhibition of active HIV-1 replication (Figure 3).

**Figure 4:**
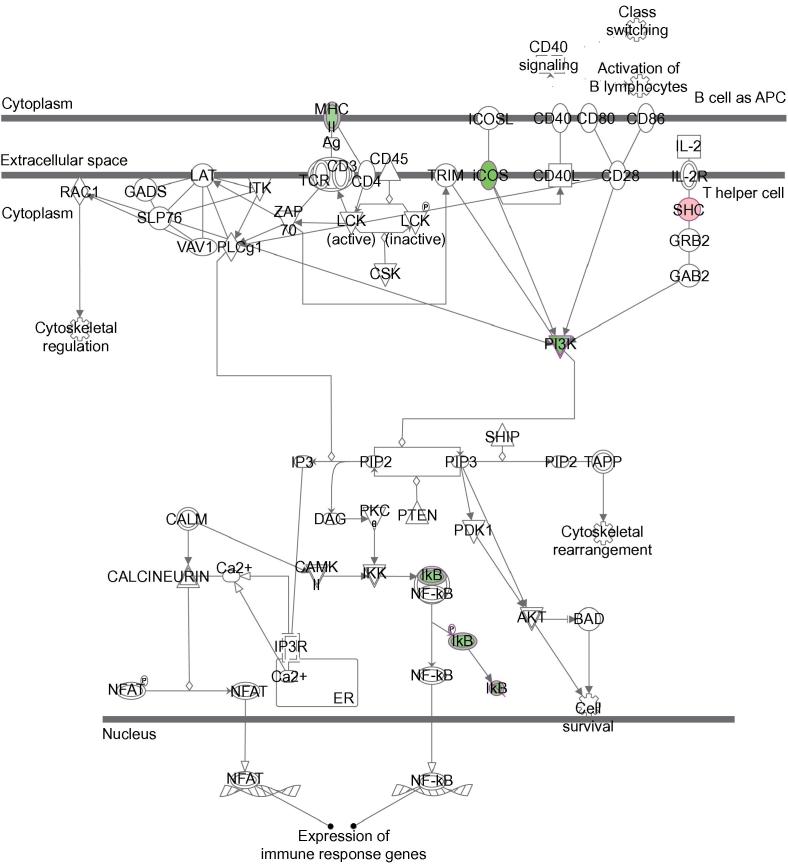
**iCOS pathway associated with HIV-1 TAR target genes** iCOS signaling in T helper cells. Proteins encoded by genes with up-regulated and down-regulated expression are highlighted in red and green, respectively.

A cellular pathway, which is particularly repressed by EBER2 HIV-1 TAR overexpression at different nodes, is the iCOS-iCOSL signaling pathway in T helper cells (Figure 4). iCOS (CD278) is expressed in activated T cells and mediates a signaling cascade via phosphatidylinositol-3-kinase (PI3K), which further downstream leads to the NF-κB-mediated expression of immune response genes. HIV-1 TAR significantly down-regulates several key players involved in this pathway, including iCOS itself, PI3K, major histocompatibility complex (MHC) class II, but also the NF-κB inhibitor IκB (Figure2C). Taken together, our results suggest a TAR regulatory function involved in the repression of the human immune response in the absence of Tat.

### A subset of HIV-1 target genes consists of HMGA1 and P-TEFb targets

**Figure 5:**
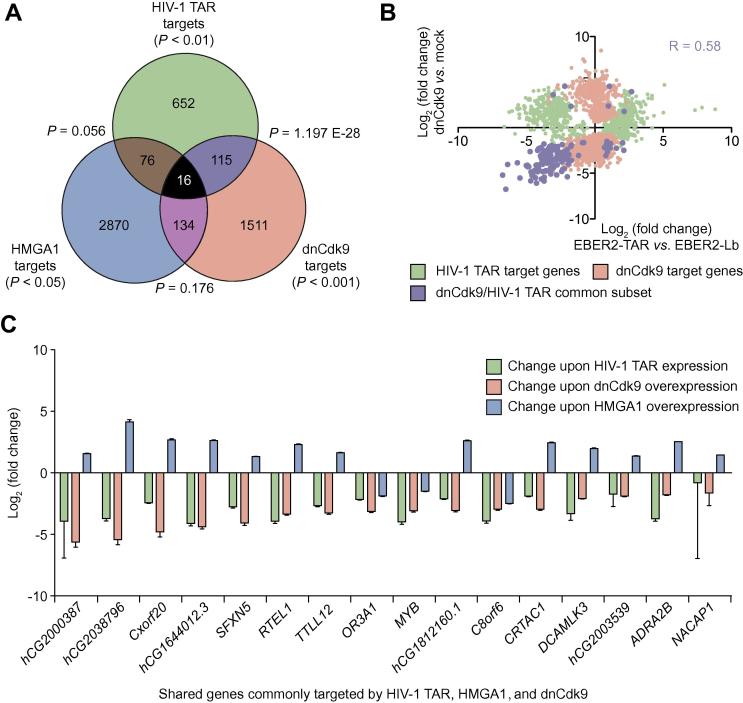
**HIV-1 TAR regulates subsets of Cdk9 and HMGA1 target genes** **A.** Venn diagram of significant genes targeted by EBER2 TAR (*P* < 0.01; green), dnCdk9 (*P* < 0.001; red), and HMGA1-Flag (*P* < 0.05; blue), respectively. The common subset of HIV-1 TAR targets and dnCdk9 targets is shown in purple, the common subset of HIV-1 TAR targets and HMGA1-Flag targets is shown in brown, the common subset of HMGA1-FLAG targets and dnCdk9 targets is shown in pink and the common subset of all three conditions is shown in black. The hypergeometric distribution *P* values for each common subset are indicated. **B.** Scatter plot of the EBER2 TAR target genes and dnCdk9 target genes as well as the common subset shown in panel A. The log_2_ fold change in gene expression upon dnCdk9 expression is indicated on the Y-axis, and the log_2_ fold change upon EBER2 TAR expression is indicated on the X-axis. The Pearson correlation coefficient (R) is displayed for the common subset. **C.** Genes of the common subset of all three conditions shown in panel A.

HIV-1 TAR has a variety of cellular interacting partners, such as TRBP, P-TEFb, and HMGA1 [7], [8], [9], [10], which all are involved in the regulation of HIV-1 TAR target genes. In a previous study, we have identified HMGA1 target genes in HEK293 cells overexpressing a FLAG-tagged version of HMGA1 [40]. Over-expression of HMGA1 results in the significant regulation of 3096 genes (*P* < 0.05), of which expression of 2159 genes is up-regulated and expression of 937 genes is down-regulated. Of these HMGA1 targets, 92 genes are also significantly regulated by overexpression of HIV-1 TAR chimera, as shown here (Figure 5A). However, the direction of such regulation is ambiguous, suggesting that interference of HMGA1 function by HIV-1 TAR, as previously demonstrated for the 7SK L2 substructure [18], to the overall TAR-mediated gene expression changes is minor. Binding of P-TEFb to HIV-1 TAR in the absence of Tat can retain P-TEFb in its inactive state [26]. Taking this into account, at least a subset of HIV-1 TAR target genes may be regulated by this HIV-1 TAR-mediated P-TEFb inactivation.

We have previously identified P-TEFb target genes by transcriptome profiling of HEK293 cells expressing a dominant negative, kinase-inactivated point-mutant of Cdk9 [40]. Such overexpression results in the significant regulation of a total of 1776 genes (*P* < 0.001), of which expression of 440 genes is up-regulated and expression of 1336 genes is down-regulated. Out of these P-TEFb targets, 131 genes are also regulated upon EBER2 HIV-1 TAR overexpression (Figure5A). Strikingly, a majority of 114 genes of this common subset are concomitantly regulated upon HIV-1 TAR expression and dnCdk9 expression (Figure5B) (hypergeometric distribution; *P* = 1.197E−28). Moreover, expression of 110 of these concomitantly-regulated genes is down-regulated in both conditions, which is in line with a HIV-1 TAR-mediated inhibition of P-TEFb.

There exists a gene-specific cooperativity of HMGA1 and P-TEFb in gene expression regulation [40]. HIV-1 TAR overexpression also targets a subset of these genes, represented as the common subset of all three conditions, EBER2 HIV-1 TAR overexpression, dnCdk9 expression, and HMGA1 overexpression (Figure5A and C). 13 out of 16 genes of this common subset are concomitantly regulated when comparing the three conditions, with expression of these genes down-regulated upon HIV-1 TAR overexpression and dnCdk9 expression and up-regulated upon HMGA1-FLAG overexpression (Figure5C). These 13 genes may thus be regulated by a HMGA1-mediated recruitment of P-TEFb and represent genes that are likely regulated by HIV-1 TAR in two ways: the TAR-mediated titration of 7SK RNA-free HMGA1, and the TAR-mediated inactivation of P-TEFb [18], [30], [40].

### The TAR-regulated P-TEFb target genes are responsive to 7SK

**Figure 6:**
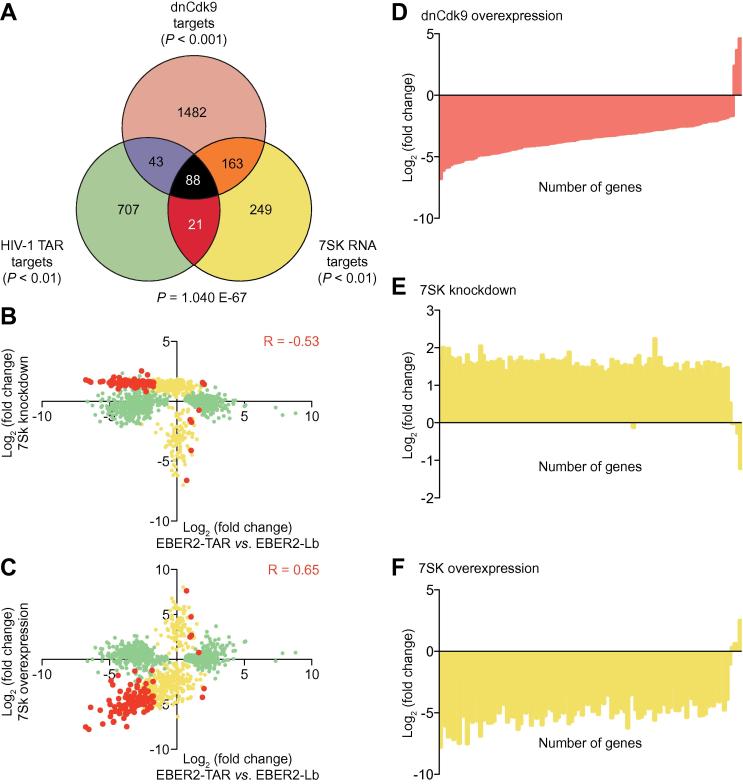
**HIV-1 TAR-regulated Cdk9 target genes are 7SK-responsive** **A.** Venn diagram of significant genes targeted by EBER2 TAR (*P* < 0.01; green), dnCdk9 (*P* < 0.001; red) and 7SK RNA (*P* < 0.01; yellow), respectively. The common subset of HIV-1 TAR targets and dnCdk9 targets is shown in purple, the common subset of HIV-1 TAR targets and 7SK RNA targets is shown in dark red, the common subset of 7SK RNA targets and dnCdk9 targets is shown in orange, and the common subset of all three conditions is shown in black. The hypergeometric distribution *P*-value for the common subset of HIV-1 TAR and 7SK RNA target genes is indicated. **B.** Scatter plot of the genes targeted by EBER2 TAR and 7SK RNA, as well as the common subset shown in panel A. The log_2_ fold change in gene expression upon 7SK knockdown is indicated on the Y-axis, and the log_2_ fold change upon EBER2 TAR expression is indicated on the X-axis. The Pearson correlation coefficient (R) is displayed for the common subset. **C.** Scatter plot of the genes targeted by EBER2 TAR and 7SK RNA, as well as the common subset shown in panel A. The log_2_ fold change in gene expression upon 7SK overexpression is indicated on the Y-axis and the log_2_ fold change upon EBER2 TAR expression is indicated on the X-axis. The Pearson correlation coefficient (R) is displayed for the common subset. The expression change of the 131 genes commonly targeted by HIV-1 TAR and dnCdk9 upon dnCdk9 overexpression (**D**), upon 7SK knockdown (**E**), and upon 7SK overexpression (**F**), respectively, are sorted from the most negative to the most positive value.

Cellular P-TEFb activity is tightly controlled by 7SK and HEXIM1, which repress Cdk9 kinase activity in a small nuclear ribonucleo protein particle (snRNP) [15], [16]. Thereby, 7SK RNA acts as a scaffold, bringing Cdk9 and Cyclin T1/T2 into close proximity of the HEXIM1 protein, which then mediates the repression of Cdk9 kinase activity. As both P-TEFb and HEXIM1 have been shown to bind HIV-1 TAR also in the absence of Tat, TAR may in this case act similar to 7SK, mediating an interaction of P-TEFb with its inhibitor HEXIM1 [26]. Thus, we compared the HIV-1 TAR target genes and the dnCdk9-regulated genes with 7SK-responsive genes. The 7SK-responsive genes were defined as genes whose expression in HEK293 cells is concordantly significantly (*P* < 0.01) regulated upon 7SK knockdown or overexpression (Figure 6A and B). As a result, we found that 109 of the EBER2 HIV-1 TAR-regulated genes are also responsive to 7SK RNA (Figure6A) (hypergeometric distribution; *P* = 1.040E−67), while 251 of the dnCdk9 target genes are also regulated by 7SK RNA. The common subset of all three conditions contains 88 genes (67%) of the dnCdk9-regulated HIV-1 TAR target genes. Furthermore, the common subset of HIV-1 TAR and 7SK target genes shows significant correlations in differential expression when compared to either 7SK knockdown (*R* =  −0.53) or overexpression (*R* = 0.65) (Figure6B and C).

To test whether the total set of HIV-1 TAR-regulated P-TEFb target genes are also regulated by the 7SK-mediated inactivation of P-TEFb, we monitored behavior of these genes in transcriptome profiles upon 7SK RNA knockdown or overexpression [40] (Figure6D–F). Out of 110 TAR-responsive P-TEFb target genes, whose expression is down-regulated upon dnCdk9 expression, expression of 108 genes (98%) are up-regulated upon 7SK knockdown (Figure6E) and concomitantly down-regulated upon *7SK* overexpression (Figure6F). These data reveal that HIV-1 TAR indeed regulates a set of classic P-TEFb target genes, most likely by repressing Cdk9 kinase activity via HEXIM1 and by interfering with cellular functions of 7SK RNA.

## Conclusions

Our study globally identifies cellular genes, which probably are highly relevant during HIV transcriptional latency in the absence of Tat. Expression of these genes is affected by HIV-1 TAR. Previous overexpression studies of HIV-1 TAR have demonstrated a significant reduction of HIV-1 replication [41], [42]. However, these studies have not investigated the effects of HIV-1 TAR expression on the host cellular gene expression. Here, by expressing a HIV-1 TAR EBER2 chimera in HEK293 cells, we observe the TAR-induced regulation of host genes involved in HIV-1 replication, which leads to a predicted significant down-regulation of HIV-1 replication (see Figure 4). Thus, the HIV-1 TAR-mediated gene regulation even in the absence of Tat may be responsible for the previously-observed reduction in HIV-1 replication. This phenomenon may be of importance for the maintenance of viral latency.

**Figure 7:**
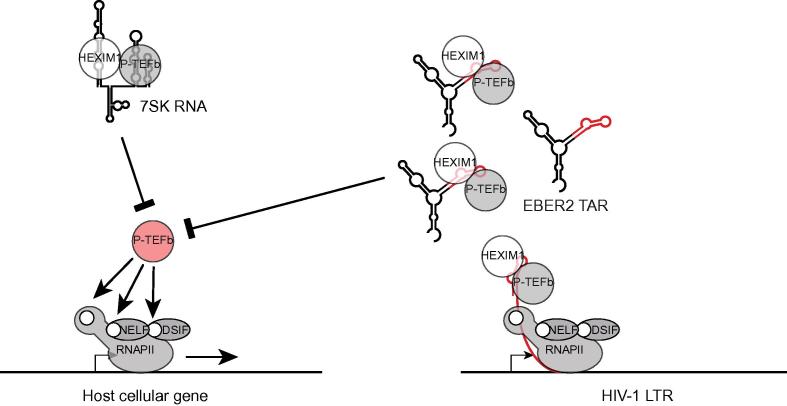
**Model for the HIV-1 TAR-mediated regulation of 7SK-responsive P-TEFb target genes** The HIV-1 TAR substructure binds to inactive, HEXIM1-associated P-TEFb in the absence of Tat – either as an RNA chimera in the context of the EBER2 RNA (EBER2 TAR) or as the nascent transcript of the HIV-1 LTR. P-TEFb is thus sequestered in its inactive form, which is endogenously stabilized by 7SK RNA. In both cases, the equilibrium between active (red) and inactive P-TEFb is shifted toward its inactive form, resulting in the repression of P-TEFb target genes. TAR, transactivating response element; HEXIM1, hexamethylene bis-acetamide-inducible protein 1; P-TEFb, positive transcription elongation factor b; Tat, transactivator of transcription; LTR, long terminal repeat; RNAPII, RNA polymerase II; DSIF, 5,6-dichloro-1-β-d-ribofuranosylbenzimidazole (DRB) sensitivity–inducing factor; NELF, negative elongation factor.

HIV-1 TAR has been shown to have various cellular binding partners and the regulation of the activity or availability of these binding partners by the overexpressed HIV-1 TAR may be the critical mechanism by which this gene expression regulation takes place. Indeed, our results suggest a TAR-mediated inactivation of P-TEFb as one potential route for the regulation of this subset of the HIV-1 TAR-responsive genes, a function that is endogenously fulfilled by 7SK RNA (Figure 7). A large proportion of these genes is implicated in central pathways involved in cellular immune responses, suggesting that HIV-1 TAR plays a role during host immune response modulation in the absence of Tat, and thus opening a path to reactivation after HIV-1 latency.

A significant set of HIV-1 TAR target genes identified here could not be assigned directly to known cellular interaction partners. For instance, TRBP has recently been shown to interact with Dicer and to contribute in that way to microRNA-mediated gene silencing [43]. Moreover, HIV-1 TAR has been suggested to sequester TRBP, resulting in an inhibition of Dicer-RISC activity [44]. Thus, a part of the HIV-1 TAR-regulated gene set may show altered expression due to an impaired Dicer activity, thus leading to significant consequences on the downstream regulatory activity. Also HIV-1 TAR itself has been reported to be processed to virus-derived microRNAs in a Tat-dependent or -independent manner [45], [46], which can alter cellular gene expression to protect against apoptosis [47] and inhibit viral replication [48]. However, as our EBER2 HIV-1 TAR chimera does not contain the full sequence encoding these microRNAs, and our approach does not include the expression of Tat, the observed gene regulatory effects cannot be a consequence of viral microRNA production. Thus, our study rather points at further, yet unidentified, specific interacting partners. Importantly, the observed significant effects of HIV-1 TAR on cellular 7SK and P-TEFb-mediated gene expression of cellular innate immune responses provide critical information on the mechanisms underlying HIV-1 latency and reactivation.

## Materials and methods

### Constructs

The EBER2 gene containing the endogenous sequence from −171 nt to +226 nt, including the complete viral promoter sequence, was inserted into the EcoRI and HindIII restriction sites of pUC18 vector (Fermentas) [29]. The resulting plasmid was then used to construct the EBER2 HIV-1 TAR RNA chimera. To obtain the EBER2 HIV-1 TAR construct, +81 nt to +140 nt sequence of wild type EBER2 RNA were replaced with +10 nt to +49 nt sequence of HIV-1 TAR RNA. To obtain the EBER2-Lb construct, the sequence from +80 nt to +145 nt of wild type EBER2 RNA was deleted as described previously [29]. Constructs used for 7SK knockdown and overexpression as well as for dnCdk9 expression have been described previously [30].

### RNA secondary structure prediction

The secondary structure of RNA molecules was calculated using RNAstructure software (Version 4.4) [49].

### Cell culture

HEK293 cells were cultured as described previously [18], [29]. Transfections were performed using FugeneHD (Roche) according to the manufacturer’s instructions. Total RNA from transfected cells was extracted using the RNeasy Midi Kit (Qiagen), as recommended by the manufacturer.

### RT-PCR

RT-PCR analyses were conducted using SuperScript™ 2 Reverse Transcriptase (Invitrogen), Lightcycler (Roche), and Fast Start DNA MasterPLUS SYBR Green I reaction mix (Roche) as recommended by the manufacturer. Primers used to quantify the expression of reference gene β-actin are 5′-TCT TCC AGC CTT CCT TCC TG-3′ (sense) and 5′-CAC GGA GTA CTT GCG CTC AG-3′ (antisense). Primers used for the quantification of the EBER2 chimeras are 5′-CCG TTG CCC TAG TGG TTT C-3′ (sense) and 5′-ACA GCG GAC AAG CCG AAT A-3′.

### Transcriptome analyses

For microarray analyses, RNA amplification, labeling, hybridization, and detection were performed following the protocols supplied by Applied Biosystems using the corresponding kits (Applied Biosystems, Prod Nos: 4339628 and 4336875). The resulting data were further analyzed as described previously [18], [34]. Briefly, the raw data were quality controlled [50], NeoNORM normalized using *k* = 0.2 [51], and then analyzed as outlined previously [52], [53], [54]. For each condition, three biological replicates were merged and compared to the merged signals of three biological replicates of the corresponding control condition. Canonical pathway enrichment studies were performed using Ingenuity Pathway Analysis® software (Ingenuity® Systems, Qiagen) as recommended by the manufacturer. Transcriptome data, annotated to the minimum information about a microarray experiment (MIAME) I + II standards, were deposited in the public database MACE (http://mace.ihes.fr) using accession numbers 2529043676 (EBER2 TAR expression), 2791359734 (dnCdk9 expression), 2655275478 (7SK overexpression), 2784969974 (7SK knockdown) and 3189818614 (HMGA1-FLAG overexpression). These data will also be made available soon through a dedicated database at 7SK.acsioma.com.

## Authors’ contributions

SE has performed the experiments, analyzed and interpreted the data, and written the manuscript. BJB has designed the study and interpreted the data. AGB has designed the study, analyzed and interpreted the data, and contributed to manuscript writing. All authors read and approved the final manuscript.

## Competing interests

All authors have declared to have no competing interests.

## Supplementary material

Supplementary Table S1 **List of genes significantly (*P* < 0.01) differentially expressed upon EBER2 HIV-1 TAR expression in HEK293 cells**

Supplementary Table S2 **List of the common target genes of EBER2 HIV-1 TAR (*P* < 0.01), dnCdk9 (*P* < 0.001), and 7SK RNA knockdown (*P* < 0.01) in HEK293 cells**

Supplementary Table S3 **List of the common target genes of EBER2 HIV-1 TAR (*P* < 0.01), dnCdk9 (*P* < 0.001), and 7SK RNA overexpression (*P* < 0.01) in HEK293 cells**
